# Who Would Taste It? Exploring Decision-Making Styles and Intention to Eat Insect-Based Food among Italian University Students

**DOI:** 10.3390/nu16203458

**Published:** 2024-10-12

**Authors:** Maria Elide Vanutelli, Roberta Adorni, Paolo Alberto Leone, Aldo Luperini, Marco D’Addario, Patrizia Steca

**Affiliations:** 1 Department of Psychology, University of Milano-Bicocca, 20126 Milan, Italy; maria.vanutelli@unimib.it (M.E.V.); marco.daddario@unimib.it (M.D.); patrizia.steca@unimib.it (P.S.); 2 Institute of Agricultural Biology and Biotechnology, National Research Council (CNR), 20133 Milan, Italy; leone@ibba.cnr.it (P.A.L.); aldo.luperini@cnr.it (A.L.)

**Keywords:** automatic attitudes, explicit attitudes, intentions, implicit association test, novel foods, insect-based food, decision-making style

## Abstract

Background: Although insect-based foods (IBFs) have been recently proposed as a way to face climate crisis and starvation, they encounter aversion from Western countries, which express fear, disgust, and high risk. The contribution of psychology research to food choices highlights how decisions are made, not only through reasoned attitudes and goal-directed behavior, but also through more automatic associations (dual-system models). Methods: In this paper, we investigated people’s dispositions towards IBFs by combining (a) explicit attitudes (as assessed via self-report scales), (b) automatic associations (as measured via indirect measures), and (c) intention to taste, and comparing different profiles based on (d) psychological factors, including decision-making style, food neophobia, and trust in science and scientist. A pilot sample of 175 Italian university students participated in the study. Results: The analyses of the general sample highlighted rather negative attitudes. The cluster analysis identified 4 decision-making profiles: ‘the gut feeling’, ‘the suspicious’, ‘the vicarious’, and ‘the mind’. It revealed more favorable opinions in ‘the mind’ profile, characterized by a rational decision-making style and high trust in science, and very aversive reactions from ‘the suspicious’ profile, characterized by high food neophobia and low trust in science. Conclusions: The results underline the importance of psychological factors in interpreting people’s reactions to IBF and changes in dietary habits based on the decision-making process. They suggest possible strategies to promote eco-friendly diets.

## 1. Introduction

### 1.1. Sustainability Goals and the Search for Alternative Protein Sources

It is no mystery that the Earth’s resources are being depleted to accommodate the demanding and unsustainable food consumption patterns of an ever-growing population. According to some authors [1] at present, none of the world’s countries have the means to meet both the growing demand for food (especially protein sources) and to ensure the protection of natural environments.

Modern agricultural and livestock practices, which are necessary for food production on a global scale, are associated with environmental issues related to over-exploitation of land and excessive water and carbon footprints [2]. Moreover, making projections to the future, experts argue that humans are already well outside what is called a ‘safe operating space’ in relation to biophysical processes [3,4]. However, this space is not only important for ensuring sustainability goals, but also health goals for the living things that inhabit these environments, including humans themselves [1]. The attempt to achieve these goals requires taking effective actions that require lower-impact and more sustainable diets, reducing consumption of animal products, or using alternative protein sources [5]. 

In recent decades, more and more methodologies (such as genetic engineering or in vitro meat production) or alternative foods have been developed, including insects. Insects have been a part of the human diet for thousands of years [6]. According to recent estimates, more than 2100 species of insects are consumed in about 140 countries. The countries with higher consumption are found in Southeastern Asia, the Americas, and Africa [7]. In some central areas of the African continent, entomophagy represents more than 50% of protein needs [8]. 

In recent years, entomophagy has attracted particular interest [9], not only because of the curiosity it generates [10], but also because insect products typically have a good nutritional profile [11], allowing us at the same time to recycle organic matter [12], which is used for their sustenance. In addition, the production process has a much lower environmental impact than other sources of animal protein in terms of greenhouse emission, land use, and water footprints [13]. However, although these products may be promising alternatives in terms of nutrition and environmental impact, their consumption remains limited, particularly in Western countries, despite the fact that some ingredients of common products have long been insect products (see, for example, the use of cochineal as a coloring agent for some alcoholic and non-alcoholic beverages and drugs) [14]. 

This skepticism is even more pronounced in the older segments of the population, who are more reluctant to try new foods [15,16,17], and also to give up taste in favor of sustainability or health motives [18,19].

For this reason, a change in the attitudes of older segments of the population seems more unfeasible, while it could be important to investigate the opinions and reactions of the younger segments, who are more open to ethnic and new foods [20,21] and could represent the most likely consumers of IBF products.

### 1.2. Attitudes towards IBFs in Western Countries

An important contribution of psychological research to entomophagy has been to investigate the relationship between psychological factors and attitudes towards IBFs. Previous studies have highlighted the role of emotional/affective variables over the development of negative attitudes, the most significant being disgust [22], food neophobia [23,24,25], and risk perception [26]. The idea of eating insects is generally accompanied by thoughts of uncertainty and fear of unhygienic food with possible negative consequences such as disease transmitting [27,28]. In their qualitative/quantitative work, Junges and colleagues [29] found that consumers with an unfavorable attitude toward insect-based foods were characterized by high food neophobia scores and suspiciousness toward these novel foods.

Previous evidence [30] has revealed that, when it comes to food-related decisions, people tend to trust those perceived as competent to make decisions for them, especially when they know little about a new technology [31]. Moreover, it has been revealed [32,33] that the trust factor toward experts or groups is a predictor of good consumer disposition toward new technologies. For example, it has been shown that trust in science and scientists can play a role in forming attitudes, but research is limited to other sustainable proteins such as cultured meat [34] or alternative production techniques, such as genetically modified organisms (GMOs) [32]. No previous studies have explored the role of trust towards IBF consumption.

### 1.3. Explicit Attitudes and Automatic Associations: Insights into IBF

Attitudes toward a particular product are an important psychological factor to consider when investigating the intention to try that product. The Theory of Planned Behavior (TPB) [35] has extensively demonstrated this link and has been successfully applied to understand entomophagy-related behaviors. Indeed, prior research has suggested that attitudes predict consumers’ intentions to try [36,37] or buy IBFs [38], highlighting how negative attitudes can predict avoidant behaviors.

Attitudes are traditionally measured by so-called direct, or explicit, measures in which participants are asked to make judgments about a topic [39]. This method, however, only partially provides the conscious point of view of a person, who may be fully or partially unaware of their attitudes or may have an ambivalent position or be influenced by perceived expectations of the research [39,40].

In fact, previous psychological research has already proved that attitudes are not always known or readily available, providing extensive and solid evidence that we do not always use analytical, rational, and conscious strategies when making a decision. Several theoretical models postulating a dual process in decision-making have been formulated in the field of cognitive and social psychology. These, which vary in their theoretical framing and constructs, are united by the idea that there are two ways in which people make decisions: a more reflective/rational one that requires more time to process information and a more impulsive and quicker one. For example, Strack and Deutsch [41] have proposed a model that distinguishes between a reflective and an impulsive system. In the former, decision-making is mediated by attitudes, reasoning, and intention, and it is a verbalizable, conscious process. In the second, behavior is enacted directly by automatic associations between categories and concepts, which are perceived as gut feeling.

To study implicit, automatic associations, so-called indirect measures are used, which generally consist of computerized tasks based on reaction times [39]. The most widely used is the Implicit Association Test (IAT) [42], which measures the strength of the associative link between positive and negative categories and attributes. The use of indirect measures improves the prediction of behavior [43,44,45], and it is particularly effective in cases where there is ambivalence [39], such as that of IBFs. In fact, food choices are also not always guided by rational considerations and are often driven by impulsive tendencies. This may be the case with IBFs, as well.

Previous entomophagy research has extensively used direct measures [26,46], while indirect measures [28,47], or both [22], have been less frequent. However, in general, both techniques have revealed unfavorable attitudes in Western countries. This seems to be primarily due to cultural beliefs that portray insects as dirty and unhealthy creatures and societies that consume them as primitive and uncivilized [48]. 

The combined use of direct/explicit and indirect/implicit measures allows for the assessment of both reflexive and more impulsive system-related processes, since they reflect the cognitive and the more spontaneous dimension of attitudes [42]. However, these two systems are not mutually exclusive, and the way people rely on one or the other when choosing or not choosing to try IBFs deserves further investigation.

### 1.4. Decision-Making Style, Attitudes, and Eating Behaviors

It has been shown that individual differences, made up of combinations of different psychological traits, could lead to different eating behaviors [49]. For example, previous research has warned that impulsivity in food-related decision-making could represent a risk factor for adopting habits that can affect our health in favor of very palatable and satisfying products, with negative consequences on diet quality [49]. The pursuit of immediate reward [50], thus, could obscure the benefits of consuming alternative foods that have long-term health and environmental benefits. However, no previous studies have explored the impact of rational vs. impulsive decision-making regarding IBFs.

Moreover, previous research has proposed that the adoption of reflexive vs. spontaneous strategies in decision-making could mediate the relationship between explicit attitudes, automatic associations, and the intention to buy [39]. One factor that has been proposed to influence the adoption of different strategies is decision-making style. Decision-making styles are considered stable characteristics [51], behavioral patterns that become recurrent [52] through the consolidation and repetition of specific reactions when a decision needs to be made [53]. In detail, Songa and Russo [39] explored if the preference for intuition or deliberation could account for participants’ preference for one of two popular soft drinks. Their findings showed that, for participants with a higher preference for an intuitive decision-making style, there was a significant increase in purchase intention when IAT scores were higher, while for participants with a less intuitive decision-making style, choices were less influenced by implicit processes. 

However, although it has been shown that the adoption of different decision-making styles can lead to different strategies in the decision-making process and to specific and diverse effects in direct and indirect measures, to the best of these authors’ knowledge, no previous study has explored the role of decision-making style on attitudes towards IBFs, nor their relationship with other possible explanatory variables of the attitudes towards IBFs, such as food neophobia and trust in science.

### 1.5. The Present Study

The aim of the present study was to investigate the propensity towards IBFs in a sample of university students. Starting from the idea that decisions derive from two parallel and distinct paths leading to automatic associations and reasoned attitudes [41], we developed a framework that involves three main outcomes:

1. Explicit attitudes, as measured by self-report scales with different semantic content. We included the following dimensions: ‘Bad’ vs. ‘Good’, ‘Risky’ vs. ‘Safe’, ‘Harmful’ vs. ‘Healthy’, and ‘Disgusting’ vs. ‘Tasty’. Based on previous literature, we expected to find:
  - Hp 1: negative attitudes, especially in the disgusting/tasty dimension.
2. Implicit attitudes, as measured by IAT. We predicted the presence of:
  - Hp 2: automatic adverse reactions toward IBFs and favorable associations for traditional foods.
3. Intention to taste, as measured regarding a specific item. We anticipated:
  - Hp 3: an average low propensity regarding taste. However, considering the age of our sample, we also expected to find several participants who were curious and inclined to taste IBF.

Moreover, we argued that individual differences could influence people’s attitudes and intention to taste IBFs. Thus, we pursued:

4. The identification of psychological profiles that could determine specific and differential dispositions towards IBFs. To achieve this, we used a person-centered approach [54], which involved categorizing individuals based on their similarities, enabling researchers to examine individuals more comprehensively than traditional approaches focused on isolated individual components.

For this stage, we included three main psychological traits. The first one was food neophobia, which has been vastly used in previous studies, as already discussed. The second is trust in science and scientists, which has been previously found to be relevant in forming attitudes toward other sustainable foods [34] or production technologies [32], but not yet in entomophagy. The third is decision-making style, which can have a role in modulating explicit/implicit attitudes but has never been explored in relation to edible insects. Given the novelty of the study, we chose the General Decision-Making Style Inventory (GDMS) [53], which measures a broad spectrum of decision-making styles. Besides the deliberative vs. intuitive continuum, which is paralleled by the rational vs. spontaneous/intuitive style [55], GDMS also includes the dependent and the avoidant scale, which can capture other aspects of the approach toward IBFs, such as the role of external guidance and social support (for the dependent style) [56], but also indecision and lack of environmental awareness (for the avoidant style) [57].

For the profiling stage we expected to find:

- Hp 3: more positive attitudes and a higher willingness to try IBFs in those participants low in food neophobia and higher in trust in science and scientists;
- Hp 4: more positive attitudes and a higher willingness to try IBFs in those participants high in the rational style. We believe that the analysis of pros and cons could more easily lead to favorable opinions and a higher intention to taste. We also predicted a worse disposition in those high in intuitive/spontaneous style, since it could be more related on emotional/instinctive components.

No specific hypotheses have been formulated for the other styles; hence, we adopted an exploratory approach. However:

- RQ: we expected to find different combinations of the profiling variables determining specific patterns in the outcomes.

## 2. Materials and Methods

### 2.1. Participants

Italian university students aged between 20 and 30 years were invited to participate in the study. Participants were recruited using a snowball sampling method. The online questionnaire was distributed via different social networks (i.e., WhatsApp, Telegram, Facebook, and LinkedIn).

A total of 175 responses were collected. Among the volunteers who completed the questionnaire, there were 93 women (53.1%), 77 men (44.0%), and five individuals (2.9%) who identified as non-binary or did not specify their gender. The participants ranged from 20 to 30 years, with a mean age of 21.85 (SD = 1.75). Regarding eating habits, 168 volunteers were omnivores or flexitarians (96.0%), while seven (4.0%) were vegetarians or vegans. Due to the low number of non-binary and vegetarian/vegan volunteers, they were not included in the statistical analyses to ensure a sufficient and homogenous sample. Moreover, seven participants produced less than 75% correct responses in the IAT and were therefore not included in the analyses. Therefore, the final sample included 158 volunteers, with 86 women (54%) and 72 men (46%) and a mean age of 21.8 years (SD = 1.56).

The sample size was calculated a priori by resorting to power analysis [58] through G*Power Software Version 3.1.9.7 [59]. Considering the exploratory nature of this pilot study, we computed the achieved power of the relevant statistical models, given *α*, sample size, and effect size.

The study was conducted following the Declaration of Helsinki and approved by the ethical committee of the University of Milano–Bicocca. Each participant provided written informed consent.

### 2.2. Procedure

Data were collected between November 2023 and June 2024. Participants were recruited mainly online by sharing the questionnaire link via WhatsApp, Telegram, Facebook, and LinkedIn.

The questionnaire was generated using Qualtrics software. It was accessible for completion online, via mobile phone or computer. Participants were initially asked to provide informed consent before processing to complete the questionnaire. Information about the study’s aim, procedure, duration, and the researchers’ contact details was provided in this section. Following that, participants were asked socio-demographic questions regarding age and gender. Additionally, participants’ psychological dispositions regarding decision-making style and trust in science and scientists were examined. This was accomplished using the General Decision-Making Style Inventory [60] and Trust in Science and Scientist Inventory [61]. Furthermore, participants were asked about their eating habits and food neophobia [62]. All variables described up to this point were treated as predictors of attitudes and intention to eat IBFs. The final part of the online questionnaire assessed the three outcome measures: explicit and automatic attitudes toward IBFs and intention to taste IBFs.

### 2.3. Measures

#### 2.3.1. Socio-Demographic Variables and Diet

Participants were asked to declare their age, gender, and eating habits. 

Eating habits: Participants had to select from 9 multiple-choice options, choosing the one that best represented their usual eating habits. The options included: ‘I regularly eat red meat, white meat, and fish’, representing an omnivorous dietary pattern; ‘I consciously reduce the meat consumption, but still eat it occasionally’; ‘I do not eat red meat, but I do eat white meat and fish’, ‘I do not eat meat, but I do eat fish’, ‘I eat organically grown, locally sourced foods, with a significant overlap with foods consumed in a vegetarian diet, but also some types of meat’, which all together indicated a flexitarian dietary pattern. Additionally, options such as ‘I do not eat meat nor fish, but I do eat eggs and dairy’, ‘I do not eat meat, fish, and eggs, but I do eat dairy’, and ‘I do not eat meat, fish, and dairy, but I do eat egg’ corresponded to a vegetarian diet. Finally, ‘I do not eat meat and fish, nor do I consume animal source products’ represented veganism, taken and modified from De Backer and Hudders [63].

#### 2.3.2. Profiling Variables

General Decision-Making Style: The General Decision-Making Style Inventory (GDMS) is a 25-item self-administered scale developed by Scott and Bruce in 1995 [53]. It is structured into five sub-scales, each representing a particular decision-making style: 1. Rational: characterized by a logical and structured approach to decision-making (e.g., ‘I double-check my information sources to be sure I have the right facts before making a decision’). 2. Intuitive: represented by a tendency to rely upon intuitions, feelings, and sensations (e.g., ‘When making a decision, I rely upon my instincts’). 3. Dependent: characterized by the need for assistance and support from others (e.g., ‘I often need the assistance of other people when making important decisions’). 4. Avoidant: represented by attempts to postpone and avoid decisions (e.g., ‘I avoid making important decisions until the pressure is on’). 5. Spontaneous: characterized by the tendency to make decisions impulsively (e.g., ‘I generally make snap decisions’). Participants express their level of agreement with each of the 25 statements on a 5-point Likert scale ranging from strongly disagree (1) to strongly agree (5). Each sub-scale score was calculated as the sum of the pertinent five items. This study used the Italian-validated version of the scale [60]. The scale showed a discrete internal consistency in the present sample (Cronbach’s alpha ranged from 0.65 for the Rational subscale to 0.84 for the Avoidant subscale).

Trust in Science and Scientists: We used the short version [61] of the Trust in Science and Scientist Inventory (TSSI) [64] to assess trust in science. The scale consists of 14 items (e.g., ‘Scientific theories are trustworthy’), which are rated on a 5-point Likert scale from strongly disagree (1) to strongly agree (5). Half of the statements are worded in reverse to measure trust in science, so responses to these statements were reversed when calculating the score. The final score was calculated as the mean score across all 14 items. A higher score indicated a greater trust in science and scientists. Given that there is no Italian version of this scale, we created an ad hoc translation of the items to measure the trust in science of the participants in our study. Two bilingual authors independently translated the text from English into Italian, and a third researcher retranslated each version of the Italian text back into English to ensure accuracy. Subsequently, all the authors discussed and agreed upon the final Italian translation. Since we used an adapted version of the scale, we conducted a Confirmatory Factor Analysis (CFA) to test the scale’s validity. The results showed that the one-factor solution sufficiently fit the data (chi-squared = 136, *df* = 73, *p* < 0.001; CFI = 0.90; SRMR = 0.065; RMSEA = 0.072, 90% CI [0.05, 0.09]). Standardized estimates were all significant (*p* < 0.001) and their values were > 0.4. Additionally, the scale demonstrated adequate internal consistency (Cronbach’s alpha = 0.85).

Food Neophobia: The level of neophobia towards new foods was assessed using the Italian version of the Food Neophobia Scale (FNS) [65], as described by Proserpio et al. in 2016 [62]. This scale measures reluctance, fear, and refusal to try new or unfamiliar foods. Respondents indicate their level of agreement with ten statements (e.g., ‘If I do not know what is in a food, I will not try it’) about foods or food situations using a 7-point Likert scale, ranging from strongly disagree (1) to strongly agree (7). Half of the statements were worded in reverse to measure food neophobia (FN), so responses to these statements were reversed when calculating the score. The FNS score was calculated as the sum of the responses to the 10 items, ranging from 10 to 70. A higher score indicates a greater level of food neophobia. In the present sample, the scale showed good internal consistency (Cronbach’s alpha = 0.87).

#### 2.3.3. Outcome Variables

Explicit attitudes toward insect-based food: Explicit attitudes toward each category of IBF (i.e., grasshopper flour, cricket burger, larvae cookies, and insect crackers) were measured by asking participants to think about it and evaluate it on a 7-point Likert scale using four pairs of adjectives within a semantic differential scale adapted from Maggino and Mola [66]. The adjectives used were ‘Bad’ vs. ‘Good’, ‘Risky’ vs. ‘Safe’, ‘Harmful’ vs. ‘Healthy’, and ‘Disgusting’ vs. ‘Tasty’. An example item was ‘What adjectives do you think are most suitable to describe grasshopper flour?’ A mean score was calculated for each of the four pairs of adjectives to explore specific attitudes toward IBFs, and a synthetic index representing the mean explicit attitude was then calculated. This synthetic index was calculated as the mean score across all items. A higher score indicated a more positive attitude toward IBFs. All the scores showed good internal consistency (Cronbach’s alpha ranged from 0.84 to 0.94).

Automatic attitudes toward insect-based food: Implicit Association Test (IAT): To identify automatic associations between IBFs, traditional foods, and positive or negative attributes, participants completed an adapted version of the Implicit Association Test (IAT) [43,67]. In this task, they were prompted to associate eight adjectives with either positive or negative valence, with eight words representing insect-based and traditional foods. The stimuli for the two target food categories consisted of four pairs of words. These pairs included grasshopper flour vs. wheat flour, cricket burgers vs. veal burgers, larvae cookies vs. rye cookies, and insect crackers vs. cereal crackers. Meanwhile, the four pairs of negative and positive attributes were the adjectives also employed for the explicit attitude measure. The underlying assumption was that individuals harboring numerous biases against IBFs would find it easier (i.e., exhibit lower response times) to associate the IBF category with negative attributes than with positive attributes. Compared with other studies using a more common version of the task (flowers vs. insects), we opted to create a more focused task that contained real traditional and insect-based foods to ensure greater ecological value.

The strength of the automatic association between the food categories and the positive or negative attributes was quantified by the D index, which is a score derived from the standardized mean difference between target-attribute pairings that are ‘inconsistent with the hypothesis’ and pairings that are ‘consistent with the hypothesis’ [67]. The D index value typically ranges from −1 to +1. A higher D index (more positive) indicates a stronger association between pairings ‘consistent with the hypothesis’ (i.e., the association between the traditional food category and positive attributes). Conversely, a negative D index suggests a stronger association between pairings ‘inconsistent with the hypothesis’ (i.e., the association between IBF category and positive attributes). A D index equal to zero indicated the absence of a significant preference for either food category. Errors were managed by requesting participants to correct their responses.

Intention to taste insect-based food in the future: Four ad-hoc items were used to ask participants about their willingness to taste four different IBFs (grasshopper flour, cricket burgers, larvae cookies, insect crackers) in the future. The full text of the items is reported below:

1. Grinding grasshoppers can produce flour for making bread, pizza, protein bars, or smoothies. Would you consider trying these grasshopper flour-based recipes in the future?
2. In some restaurants, it is possible to taste burgers made with cricket flour. Would you like to try them in the future?
3. There are cookies on the market produced using dried moth larvae. Would you like to try them in the future?
4. It is already possible to buy crackers made from dried insects. Would you like to try them in the future?

The response options range from 1: ‘extremely unlikely’ to 10: ‘extremely likely’. The responses were analyzed separately, and a synthetic index representing the mean intention to taste IBF was calculated. This synthetic index was calculated as the mean score across all four items. A higher score indicates a greater intention to taste IBF in the future. The synthetic index showed good internal consistency (Cronbach’s alpha = 0.96).

### 2.4. Data Analysis

To answer the research questions, the following analyses were conducted: 

#### 2.4.1. Preliminary Analyses

Preliminary analyses were performed on the dataset to verify data normality and the internal consistency of the psychological scales. Cronbach’s alpha was calculated to estimate the internal consistency of the psychological scales [68]. Because an adapted version of the Trust in Science and Scientists scale was used, Confirmatory Factor Analysis (CFA) was performed. Hu and Bentler’s guidelines [69] for several fit indices were employed to decide if the expected models were consistent with the data. A good model yields a nonsignificant chi-square statistic, a comparative fit index (CFI) higher than 0.90, and a weighted root-mean-square residual (SRMR) lower than 1.0. Values close to 0.06 for the RMSEA indicate a good fit; between 0.06 and 0.08, a moderate fit, and values larger than 0.10 indicate a poor fit.

#### 2.4.2. Identification of Psychological Profiles

1. Cluster analyses were performed on the continuous scores of the psychological traits (FNS, TSSI, GDMS), following the recommendations of Bergman and colleagues [70]. First, all variables were standardized. Additionally, a residue analysis was conducted (average squared Euclidean distance—ASED—less than 0.5). Ten multivariate outliers were identified (6.3% of the sample) and removed from the subsequent analyses. A two-step clustering procedure was used, which combined Ward’s hierarchical and nonhierarchical k-means methods. In the hierarchical method, different solutions were explored based on the magnitude of the change in the explained error sum of squares percentage (%EESS) value between adjacent cluster solutions. Subsequently, each solution was employed as the initial cluster center for a nonhierarchical k-means clustering procedure.
2. Descriptive statistics were calculated on all profiling variables.
3. Differences in age and gender distribution were investigated. For gender analysis, χ^2^ test was run on cluster and gender variables. For age analysis, a univariate analysis of variance (ANOVA) was performed with age as a dependent variable, and cluster as an independent variable.

#### 2.4.3. Differences between Clusters on Outcome Variables

1. Descriptive statistics were calculated on the outcome variables.
2. Three separate univariate ANOVAs were conducted using mean explicit attitudes, automatic attitudes, and the intention to taste IBFs as dependent variables. The independent variable in each analysis was cluster. Post hoc Tukey tests were used for comparisons when variances were equal, while the Games–Howell method was used when variances were unequal. Before conducting the analyses, the normal distribution of the variables was confirmed through assessments of skewness and kurtosis, and the homogeneity of variances was evaluated using Levene’s test.

All statistical tests were two-tailed, and a *p* ≤ 0.05 was considered statistically significant. Analyses were performed using IBM SPSS Statistics, version 29 (SPSS, Chicago, IL, USA) and Jamovi (Version 2.2.5, The Jamovi project, 2021, retrieved from https://www.jamovi.org, accessed on 1 August 2024). The statistical package ROPstat [71] was used for typological analyses.

The dataset that produced the results presented and discussed in the article is provided as Supplementary Material.

## 3. Results

### 3.1. Preliminary Analyses

The normal distribution of the data was tested by calculating skewness and kurtosis indices; the recommended range of ±2 and ±7 was considered for normality, respectively [72]. All variables were normally distributed. All scales had moderate to good fit.

### 3.2. Identification of Psychological Profiles

#### 3.2.1. Cluster Analyses

After analyzing the scree-type plot displaying the change in %EESS by cluster solutions and considering the magnitude of the change in the %EESS values, we decided to retain the solution involving four clusters (%EESS = 35.7). Given that the study is a pilot with a relatively small sample size, we concluded the process by extracting four clusters, that ensured a numerosity of at least 30 cases per cluster (at least 20% of cases per cluster). Participants were well distributed in the four clusters (see Table 1).

**Figure 1:**
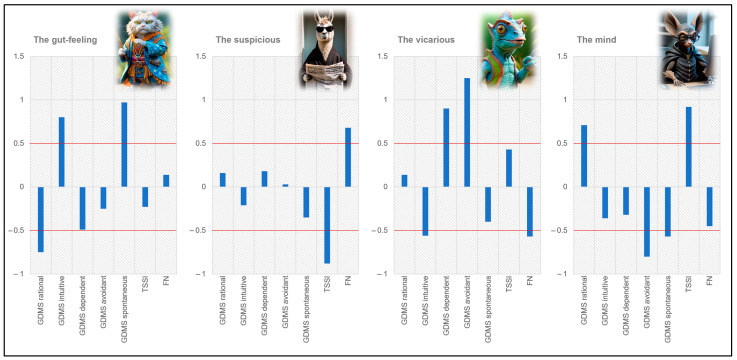
Z scores of psychological traits for the 4 clusters (n = 148).

Figure 1 presents the cluster solution. The y axis represents Z scores. Because the clusters were defined using Z scores for the total sample, each cluster’s mean Z scores indicate the distance between the cluster means and the total sample’s standardized mean. In other words, a Z score between −0.5 and +0.5 denoted an average value (i.e., the ‘average participant’ psychological traits). A Z score over +0.5 denoted values above the sample mean.

Cluster 1, ‘the gut feeling’, was characterized by a more intuitive/spontaneous and less rational decision-making style than the average sample. We chose the cat for its avatar, as it is an extremely sensitive and instinctive animal.

Cluster 2, ‘the suspicious’, was characterized by high food neophobia and low trust in science and scientists. We chose the llama for its avatar because it spits when it feels threatened. 

Cluster 3, ‘the vicarious’, was characterized by a more dependent/avoidant and less intuitive decision-making style, with low food neophobia as well. We chose the chameleon for its avatar to represent the vicarious profile’s tendency to mimic what others do and to go along with the environment.

Cluster 4, ‘the mind’, was characterized by high trust in science and scientists and a decision-making style that is more rational than avoidant/spontaneous. We chose the bat for its avatar, since it is technological (it possesses biosonar).

#### 3.2.2. Descriptive Statistics

Descriptive data are reported in Table 1 with cluster number, cluster size, mean age, and gender frequency, along with data related to all the psychological scales.

**Table 1:** Sociodemographic description and mean psychological traits for the identified clusters (n = 148).

							Mean (SD)			
Cluster	n (%)	Mean Age (SD)	n Male (%)	GDMSr	GDMSi	GDMSd	GDMSa	GDMSs	TSSI	FNS
1	46 (31%)	21.7 (1.37)	19 (41.3%)	18.8 (1.48)	18.5 (1.82)	16.9 (3.02)	12.7 (3.37)	15.2 (2.75)	4.10 (0.31)	31.7 (9.28)
2	38 (25.7%)	21.5 (0.95)	12 (31.6%)	20.5 (1.48)	15.9 (1.62)	19.2 (2.80)	13.9 (3.33)	11.3 (2.01)	3.84 (0.26)	37.2 (9.07)
3	30 (20.3%)	21.4 (1.04)	13 (43.3%)	20.5 (1.74)	15.0 (2.78)	21.5 (2.69)	19.2 (3.09)	11.1 (2.10)	4.37 (0.35)	24.4 (8.90)
4	34 (23%)	22.4 (2.22)	21 (61.8%)	21.6 (1.60)	16.0 (2.46)	17.5 (2.80)	10.4 (2.41)	10.6 (1.84)	4.57 (0.29)	25.6 (8.57)

Note. GDMS = General Decision-Making Style Inventory (range 5–25); GDMSr = rational; GDMSi = intuitive; GDMSd = dependent; GDMSa = avoidant; GDMSs = spontaneous; TSSI = Trust in Science and Scientist Inventory (range 1–5); FNS = Food Neophobia Scale (range 10–70).

#### 3.2.3. Gender Differences

The clusters did not differ in terms of age [Welch’s F (3, 75.2) = 2.07; *p* = 0.11; Levene’s test (3, 144) = 4.49; *p* < 0.01] or gender [χ^2^ (3, n = 148) = 6.88; *p* = 0.076]. However, although the analysis was not statistically significant, it is possible to appreciate from a qualitative point of view that the frequency of men was higher in Cluster 4 (‘the mind’), while the frequency of women was higher in all the other cluster, especially Cluster 2 (‘the suspicious’).

### 3.3. Differences between Clusters on Outcome Variables

#### 3.3.1. Explicit Attitudes towards IBFs

In general, participants showed a more negative attitude toward IBFs when rated along the disgusting vs. tasty scale (M = 3.35; SD = 1.37), followed by the bad vs. good scale (M = 3.63; SD = 1.42), and the harmful vs. healthy scale (M = 4.23; SD = 1.60). The most positive attitude was observed on the risky vs. safe scale (M = 4.42; SD = 1.69).

**Figure 2:**
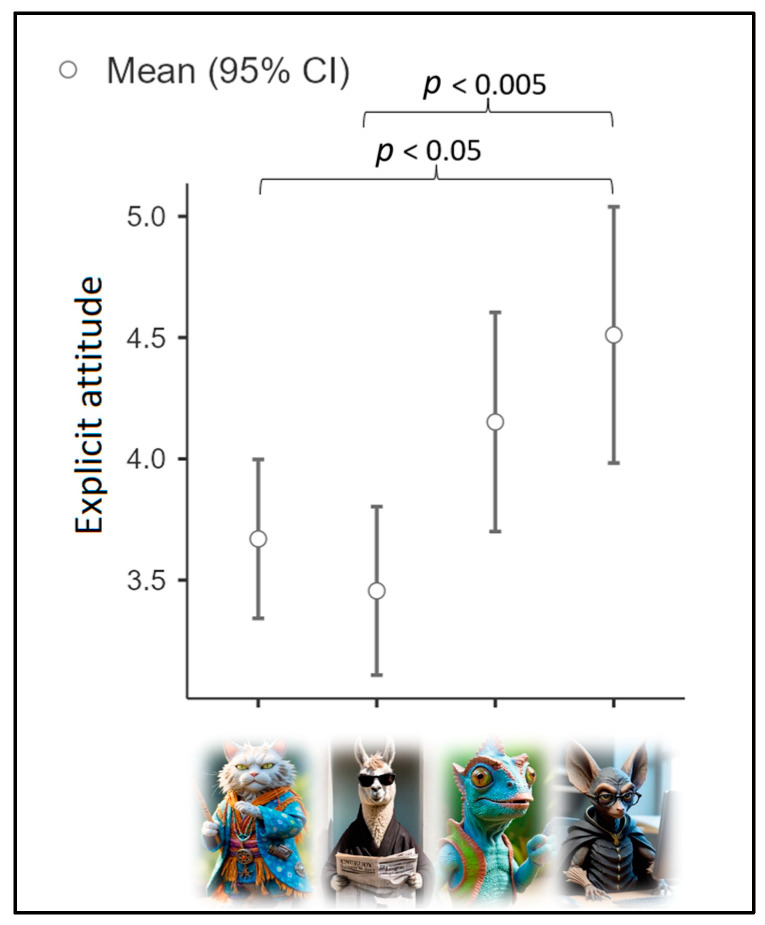
Mean explicit attitudes based on cluster.

The mean explicit attitude (M = 3.91; SD = 1.27) was used as a dependent variable in the first ANOVA, with cluster as an independent variable. Assumption checks suggested that the group variances were homogeneous [Levene’s test (3, 144) = 1.01; *p* = 0.39]. Results (see Figure 2) highlighted a significant effect of group [Fisher’s F (3, 144) = 5.5; *p* < 0.005; η^2^p = 0.1; achieved power = 0.94]. Cluster 4 declared the more favorable attitude toward IBFs (M = 4.51; SD = 1.51), followed by Cluster 3 (M = 4.15; SD = 1.21) and Cluster 1 (M = 3.67; SD = 1.10). Cluster 2 declared the most negative attitude toward IBFs (M = 3.46; SD = 1.06). Post hoc comparisons showed that the difference between Cluster 1 and Cluster 4 was statistically significant (*p* < 0.05), as well as the difference between Cluster 2 and Cluster 4 (*p* < 0.005).

#### 3.3.2. Automatic Attitudes towards IBFs

**Figure 3:**
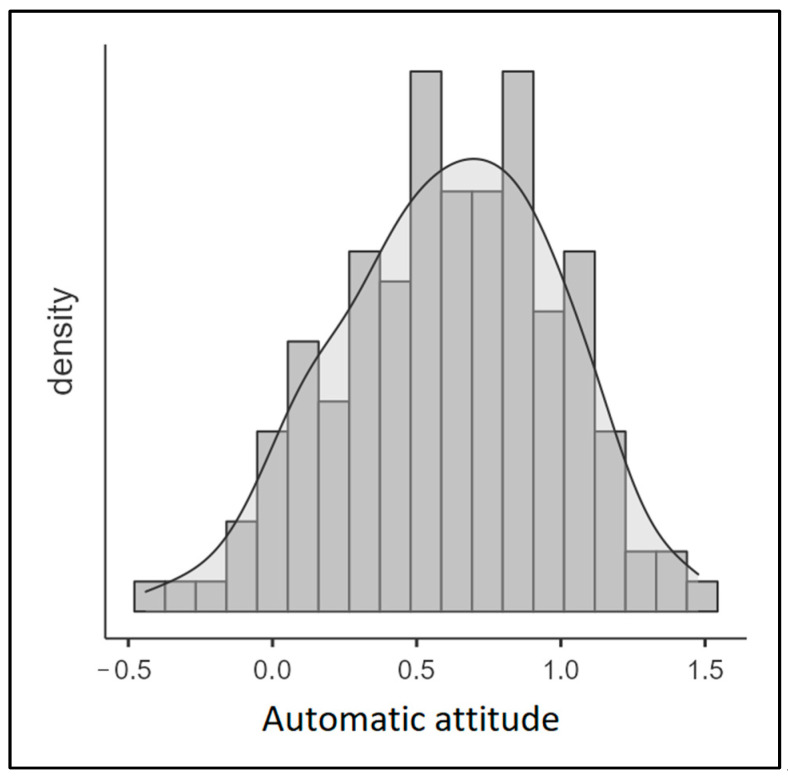
D score distribution (n = 148).

The mean D score for the whole sample was 0.61 (SD = 0.38) and had a normal distribution (skewness = −0.20; kurtosis = −0.38, Figure 3). As the procedure section outlines, a higher D score indicates a stronger association between positive attributes and traditional food. In contrast, negative D scores indicate a stronger association between positive attributes and IBF. Therefore, the results suggested that the participants displayed a more favorable automatic attitude toward traditional food than toward IBFs.

**Figure 4:**
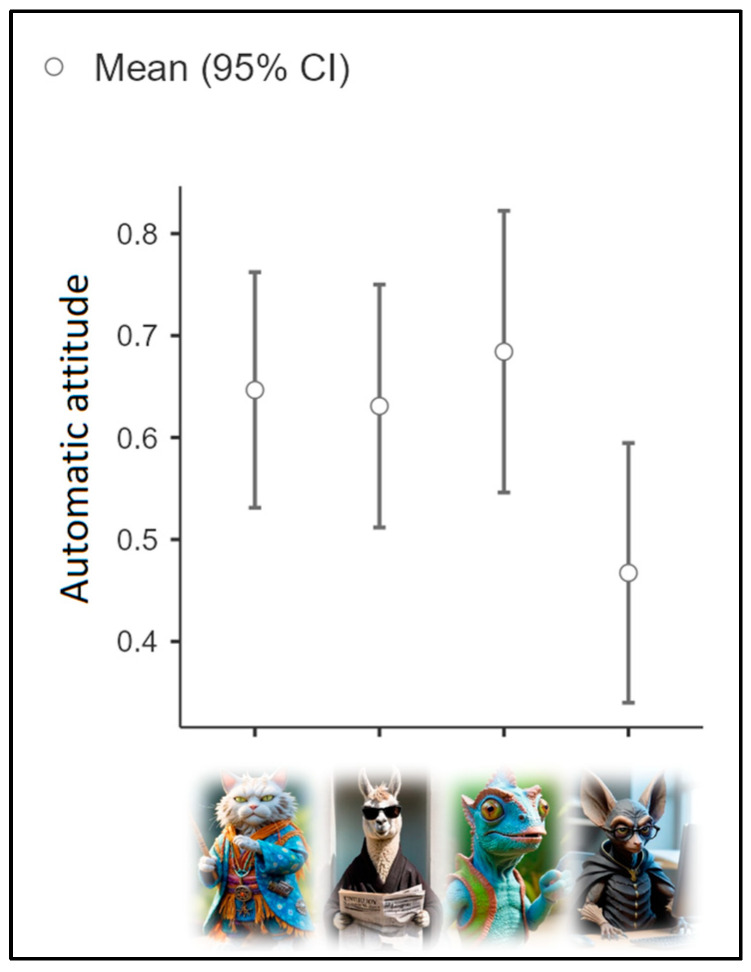
Mean automatic attitudes based on cluster.

The second ANOVA considered the automatic attitude toward IBFs as the dependent variable and the division into clusters as the independent variable. Assumption checks suggested that the group variances were homogeneous [Levene’s test (3, 144) = 0.09; *p* = 0.97]. Results (see Figure 4) did not evidence a significant group difference (*p* = 0.086). However, from a descriptive perspective, Cluster 4 exhibited a more favorable automatic attitude toward IBFs (M = 0.47; SD = 0.37), followed by Cluster 2 (M = 0.63; SD = 0.36) and Cluster 1 (M = 0.65; SD = 0.39). Cluster 3 displayed the most negative automatic attitude toward IBF (M = 0.68; SD = 0.37).

#### 3.3.3. Intention to Taste IBFs

Descriptive statistics suggested that, in general, participants had little intention of trying cookies made with moth larvae (M = 4.85; SD = 3.17), followed by insect crackers (M = 5.77; M = 2.97) and cricket burgers (M = 6.34; SD = 2.85). They were more inclined to taste products made from grasshopper flour (M = 6.74; SD = 2.75).

**Figure 5:**
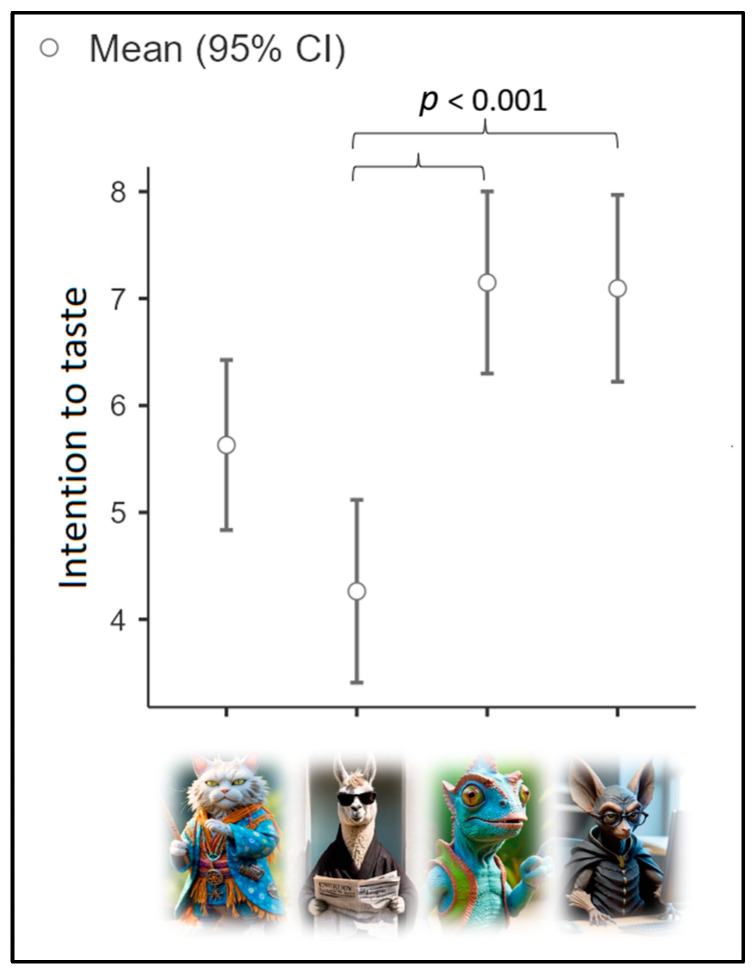
Mean intention to taste IBFs based on cluster.

We used the mean intention to taste IBF (M = 5.92; SD = 2.77) as the dependent variable for the third ANOVA, with cluster as the independent variable. Assumption checks suggested that the group variances were homogeneous [Levene’s test (3, 144) = 0.97; *p* = 0.41]. Results (see Figure 5) highlighted a significant effect of group [Fisher’s F (3, 144) = 10.53; *p* < 0.001; η^2^p = 0.18; achieved power = 0.998]. Cluster 3 declared the highest intention to taste IBFs (M = 7.15; SD = 2.28), followed by Cluster 4 (M = 7.1; SD = 2.50) and Cluster 1 (M = 5.63; SD = 2.68). Cluster 2 declared the lowest intention to taste IBFs (M = 4.26; SD = 2.60). Post hoc comparisons showed that the difference between Cluster 2 and Cluster 4 was statistically significant (*p* < 0.001), as was the difference between Cluster 2 vs. Cluster 3 (*p* < 0.001).

## 4. Discussion

The purpose of the present study was to investigate the role of different psychological profiles in relation to a novel and rather controversial behavior in Western countries: eating insect products. The interest was particularly in exploring the effect on both reflexive and impulsive processes that may be associated with the enactment of the actual behavior, namely, explicit attitudes and automatic associations. Then, we profiled participants into separate clusters and compared them in terms of the different outcomes.

The results identified some interesting data. First, considering the whole sample, unfavorable attitudes toward IBFs emerged, consistent with hypotheses and previous literature. This trend manifested in all three outcomes. For explicit attitudes, the lowest scores were given along the disgusting vs. tasty scale, with a mean of 3.35, while the least problematic scores were given along the risky vs. safe scale, with a mean of 4.42. As discussed in the introduction, both disgust [22] and risk perception [26] are important resistance factors in IBF consumption, but disgust is probably the biggest obstacle [28].

The negative attitude also emerged at the automatic level, as can be appreciated from the d index. This, 0.61, indicates the presence of significantly more favorable automatic associations toward traditional food.

About the intention to taste, the participants in our sample expressed on average an intention of 5.92 out of 10 to taste IBFs. This seems a bit more favorable than what Roma and colleagues found [16] in another Italian sample. In fact, the researchers found only 22.9% of their sample were willing to taste IBFs. This can be explained by taking into consideration the sample’s age. In fact, previous work has already underlined that age is negatively related to the acceptance of insects as food [15,16,17]. Our sample was composed only of young adults, while Roma and colleagues’ sample ranged from 18 to 81 years old. Their results showed that the consumers who strongly rejected IBFs in any form or preparation were the oldest [16]. 

Moreover, the product considered least tempting in our sample was moth larva cookies, while grasshopper flour was the one considered most appealing. In the work by Roma and colleagues [16], the percentage of participants willing to taste was significantly higher (16.8%) when it came to products where insects were not visible, compared to the case where insects were visible (2.3%). In a large study involving 13 countries worldwide, Castro and Chambers [73] asked participants what the main psychological or sensory reasons were for choosing whether or not to taste certain products. Participants from all nations cited appearance as the main reason, making explicit that it is important that you do not see bits of insects in the food. A preparation made from insect flour, therefore, seems to be the one that generates the least adverse reactions. In our case, probably the word ‘larvae’ accentuated the feeling of disgust, since larvae have been found as being even more problematic than adult insects [74]. 

However, what do individual differences tell us about outcome variables? We identified four main profiles by performing a cluster analysis, including factors that might contribute to the decision-making process in different ways. These comprised both well-known factors in the literature, such as food neophobia, and variables new to the topic area, such as trust in science and decision-making style. The four main profiles were ‘the gut feeling’, ‘the suspicious’, ‘the vicarious’, and ‘the mind’. After identifying the psychological profiles, we explored the differences on the main outcomes, namely explicit and automatic attitudes, and intention to taste IBFs.

- The ‘gut feeling’ profile is mainly characterized by a decision-making style with a combination of spontaneous and intuitive and not at all rational. We are, therefore, faced with people who make decisions solely based on their feelings and emotions, in a very quick way. The cluster is characterized by rather negative attitudes, both implicit and explicit, and a modest intention to taste. Not surprisingly, avoidance of analytical reasoning regarding pros and cons, and trust in one’s own instincts, can lead to avoidance behavior toward IBFs. In fact, several previous contributions have highlighted the crucial role of emotional aspects, and particularly of disgust, toward IBFs. For example, in the previously cited work by La Barbera and colleagues [28], food neophobia and the emotion of disgust were found to negatively and independently affect the intention to eat IBFs. The explanatory power of disgust was even greater. This important finding underscores how, although the two constructs may be similar, they do not overlap and thus may contribute specifically and differentially to the outcomes. The modest declared intention to taste may be more determined by the tendency to try and not back down, but it does not seem very promising, since it is not supported by favorable attitudes. For the ‘gut feeling’ profile, we can speculate that the emotion of disgust might be one of the determinants of aversion to IBF, and that a more intuitive/impulsive system for decision-making could be adopted. Although not statistically significant, this profile was the one with the highest percentage of women. This finding is also reflected in the literature, as it has been found that women are generally more reluctant to accept IBFs and report higher FN and disgust scores [17,75,76].
- ‘The suspicious’ profile finds support in previous literature, as it is characterized by high food neophobia, negative attitudes, and low intention to taste IBFs. This profile is similar to one of the two profiles identified by Junges and colleagues [29] in their qualitative/quantitative work. The segments identified were ‘consumers with a favorable attitude toward insect-based foods’ and ‘consumers with an unfavorable attitude toward insect-based foods.’ The main characteristic of people belonging to the second segment, in addition to negative attitudes and low intention to eat IBFs, were high food neophobia scores and suspiciousness toward these novel foods. The negative relationship between food neophobia and willingness toward IBF has already been widely confirmed in the literature [23,24,25]. In Verbeke’s work [17], it was found that the increase of just one unit in food neophobia scores led to an 84% decrease in the likelihood of being ready to adopt a diet that includes IBFs. A very interesting perspective is offered by the work of Jaeger and colleagues [77], which showed that people with higher FN scores rated the emotional impact of food more negatively and with greater arousal. ‘The suspicious’ profile, moreover, is characterized by very low trust in science. This result, which has no previous findings in the entomophagy literature, is in line with our hypotheses, as it was, instead, identified with other sustainable foods. In their work investigating openness to try cultured meat, Lewish and Riefler [34] found that distrust of scientists was negatively related to behavioral intention. Similar findings emerged on the acceptance of genetically modified foods [32].
- ‘The vicarious’ profile is characterized by a fair overt disposition toward IBF in terms of both explicit attitudes and intention to taste. However, this good disposition is not matched by automatic attitudes and the scores are comparable to those of the two less favorable profiles. How can this discrepancy be explained? The cluster is characterized by the concurrence of two decision-making styles: avoidant and dependent. The avoidant style is prone to postponing any decision and correlates negatively with rationality in decision-making [56]. The dependent needs confirmation and seeks external references to make decisions, such as advice from trusted people, but also from what authorities suggest. More interestingly, both the avoidant and dependent profiles are positively associated with indecisiveness, as opposed to the rational style [56]. At the same time, they present low food neophobia. This aspect is very important, since it indicates how low food neophobia is not sufficient to develop totally favorable dispositions, as already argued, nor to explain this ambivalence in cluster 3. It is therefore possible to hypothesize that the indecisiveness that characterizes both these styles may have led people to respond relatively positively to explicit questions, either because they did not have to think too much (avoidant) or because of social desirability (dependent), but still manifest a low propensity toward a more automatic level. In this case, it is possible to hypothesize a conflict between the two systems.
- Finally, ‘the mind’ profile, characterized by a rational decision-making style and high trust in science, has more positive attitudes than the other profiles toward IBFs and a higher intention to taste them. These characteristics are partially reflected in the literature. In a previous study, Vernau and colleagues [45] investigated the intention of an Italian and a Danish sample to include IBFs in their diet by performing market segmentation based on their scores on the Food-Related Lifestyle Scale. Although they used different tools than those employed in the present work, the researchers identified a ‘rational food consumer’ profile, corresponding to an informed person who gathers information about the products they buy and considers multiple factors at once when shopping. Again, this profile was the one that declared a more favorable intention. Indeed, the main characteristics of the rational decision maker [56] involve a logical evaluation of possible alternatives and a meticulous search for information, as also confirmed by eye-tracking data on product labels [78]. In addition, a positive correlation between rational style and cognitive engagement has previously been revealed [56]. A more positive propensity toward IBFs is not only explicitly stated by ‘the mind’ cluster, but also emerges from the reaction times of the IAT, albeit not statistically significantly. This profile is the only one with a higher percentage of men respondents. This result has a basis in previous literature, since men have been found to be more accepting of IBFs than women [76,79]. An Italian study [80] demonstrated that men were 2.55 times more likely to be open to insect consumption. However, the analysis of gender differences in previous research has produced mixed results [81,82] and so deserves a more thorough exploration in future studies.

The absence of a significant effect in d indices may still be a meaningful finding: as differences emerge about what participants overtly state, the absence of differences in automatic reactions may indicate that while there are more favorable profiles to tasting and consuming IBFs (such as ‘the mind’ and ‘the vicarious’), there is still much resistance on an implicit level that warrants future investigation. It is possible to hypothesize that, while from a cognitive point of view, it is possible to identify good and convincing arguments that dispose people well to IBFs, favorable impulsive reactions to this novelty require more time and familiarity. Indeed, it has been hypothesized that automatic attitudes change more slowly over time than explicit ones [83]. The unfavorable position towards IBFs, then, is very pronounced, not only because they are foods produced through the processing of animals that in Western culture are considered gross, but also because they are products that people have never or rarely encountered. 

The present work is not without limitations. First, the selection of psychological factors for profiling can certainly not be considered exhaustive, as many other aspects may contribute to modulate intention-to-taste behavior. Some possible explanations, such as those related to disgust for ‘the gut feeling’ profile or social desirability for ‘the vicarious’ profile, have not been measured with specific instruments, but can only be hypothesized and explored in future research.

Moreover, since this was a pilot study, some results of interest did not reach statistical significance due to the small sample and the large number of variables involved. Starting from these initial findings, the future goal is to test our hypotheses in a larger and more representative sample of the general population. 

This work has several strengths and innovations. First, it represents the first example in which decision-making style is integrated into the entomophagy literature. Second, the person-centered statistical approach enabled us to comprehend how particular combinations of psychological dispositions lead to different outcomes. This approach has the potential to offer deeper insights into how individuals form their attitudes towards IBF and, as a result, their behaviors in real-life scenarios.

Also, the simultaneous measurement of explicit and automatic attitudes may give way to future conversations on how these aspects interact with each other at different levels.

## 5. Conclusions

The purpose of this research was to investigate how attitudes and intention to taste IBF could vary according to certain psychological traits. Specifically, the role of more reflexive/cognitive and more associative/automatic processes on the propensity toward IBFs was examined. 

A person-centered approach was adopted and four different profiles were identified: The gut feeling, the suspicious, the vicarious, and the mind decision-makers. These four clusters showed unique and specific combinations of decision-making style, food neophobia, and trust in science and revealed the role of these psychological factors in food-related attitudes and intentions, helping to expand current literature on processes and psychological factors affecting lifestyle decision-making. For example, they provide new insights to the field of entomophagy research by describing people’s reactions to new food proposals. In light of our results, it will be possible to understand more deeply people’s openness to these products as an alternative to more traditional animal-based protein sources. Finally, our findings may support the development of strategies to provide adequate information and support decision-making, taking into account of different psychological profiles.

## Data Availability

The original contributions presented in the study are included in the article/Supplementary Material, further inquiries can be directed to the corresponding author/s.

## Supplementary Materials

The following supporting information can be downloaded at: https://www.mdpi.com/article/10.3390/nu16203458/s1.
